# Effective combination of isolated symptom variables to help stratifying acute undifferentiated chest pain in the emergency department

**DOI:** 10.1002/clc.23170

**Published:** 2019-03-19

**Authors:** Wen Zheng, Jingjing Ma, Shuo Wu, Guangmei Wang, He Zhang, Jiaqi Zheng, Feng Xu, Jiali Wang, Yuguo Chen

**Affiliations:** ^1^ Department of Emergency and Chest Pain Center Qilu Hospital, Shandong University Jinan China; ^2^ Key Laboratory of Emergency and Critical Care Medicine of Shandong Province Qilu Hospital, Shandong University Jinan China; ^3^ Institute of Emergency and Critical Care Medicine Shandong University Jinan China; ^4^ Key Laboratory of Cardiovascular Remodeling & Function Research, Chinese Ministry of Education & Chinese Ministry of Public Health Qilu Hospital, Shandong University Jinan China

**Keywords:** chest pain, emergency department, score, symptom

## Abstract

**Background:**

Symptom is still indispensable for the stratification of chest pain in the emergency department. However, it is a sophisticated aggregation of several aspects of characteristics and effective combination of those variables remains deficient. We aimed to develop and validate a chest pain symptom score (CPSS) to address this issue.

**Hypothesis:**

The CPSS may help stratifying acute undifferentiated chest pain in ED.

**Methods:**

Patients with non‐ST segment elevation chest pain and negative cardiac troponin (cTn) over 3 hours after symptom onset were consecutively recruited as the derivation cohort. Logistic regression analyses identified statistical predictors from all symptom aspects for 30‐day acute myocardial infarction (AMI) or death. The performance of CPSS was compared with the symptom classification methods of the history variable in the history, electrocardiograph, age, risk factors, troponin (HEART) score. This new model was validated in a separated cohort of patients with negative cTn within 3 hours.

**Results:**

Seven predictors in four aspects of chest pain symptom were identified. The CPSS was an independent predictor for 30‐day AMI or death (*P* < 0.001). In the derivation (n = 1434) and validation (n = 976) cohorts, the expected and observed event rates were well calibrated (Hosmer–Lemeshow test *P* > 0.30), and the c‐statistics of CPSS were 0.72 and 0.73, separately, significantly better than the previous history classifications in HEART score (*P* < 0.001). Replacing the history variable with the CPSS improved the discrimination and risk classification of HEART score significantly (*P* < 0.001).

**Conclusions:**

The effective combination of isolated variables was meaningful to make the most stratification value of symptoms. This model should be considered as part of a comprehensive strategy for chest pain triage.

## 1 INTRODUCTION

Chest pain and related symptoms rank the top reasons for visits to emergency department (ED) all over the world,^1^, ^2^ which are extremely heterogeneous with a wide spectrum of conditions ranging from lethal diseases such as acute myocardial infarction (AMI) to minor acute problems such as intercostal neuralgia. The inappropriate discharge of high‐risk patients with AMI from the EDs would lead to nearly two times mortality when compared with admitted patients.^3^ However, the majority of undifferentiated acute chest pain are low risk and not myocardial ischemic related, not requiring unnecessary further invasive tests or admission.^4^, ^5^ Therefore, rapid risk stratification models for chest pain patients at the EDs are recommended, in order to guarantee the early treatment and management of AMI patients and safe discharge of low‐risk patients.^4^, ^6^

Characteristics of acute chest pain symptom are important components in the prediction models for stratification of acute chest pain patients in ED.^7^, ^8^, ^9^ Although sensitivity and specificity of symptom elements are not excellent enough,^10^ the roles of these variables to diagnose AMI and stratify chest pain are indispensable,^11^ because the probability of AMI is dependent on not only the cardiac troponin (cTn) value but also pretest probability.^12^ As a simple and economic diagnostic tool, detailed description of chest pain symptom may provide useful information to estimate pretesting probability for scrupulous interpretation of cTn.^12^

Normally, chest pain symptom is a sophisticated aggregation of several sections of variables to describe the characteristics from different aspects, including character, location, radiation, severity, time course, associated symptoms, precipitating factors, and relieving factors. However, in some predict scores, some symptom components were assessed with electrocardiograph (ECG) and cTn in the logistic regression as isolated variables,^8^, ^9^ which may miss the importance stratifying power of organically integrated symptom features. In other scores, like the history, ECG, age, risk factors, troponin (HEART) score, although combination of symptom variables had been taken into consideration as the history, suspicious components for typical angina were still not clearly stated and not assessed systematically.^7^ Besides, in spite of different symptom classification methods have been attempted and reported previously, the performance is ambiguous.^13^, ^14^

As we all know, cTn can be detected firstly 3 to 4 hours after AMI occurs due to its kinetic profile of releasing. However, negative cTn over 3 hours after symptom onset was not safe enough to rule out events.^15^ Therefore, we aimed to develop a chest pain symptom score (CPSS), using statistical combination of acute clinical features for predicting 30‐day AMI or death in acute chest pain patients presenting to the ED with negative initial cTn over 3 hours after symptom onset. And we externally validated the CPSS in a simultaneously recruited cohort of chest pain patients with negative initial cTn within 3 hours after symptom onset.

## 2 METHODS

### 2.1 Study design

This is a prospective cohort study of acute non‐ST segment elevation chest pain in the urban ED of the Qilu Hospital of Shandong University (a university‐affiliated teaching hospital), and in the rural ED of the People's Hospital of Wenshang County from 24 August 2015 to 30 September 2017. The derivation cohort consisted of patients enrolled consecutively with negative initial cTn over 3 hours after symptom onset. And the validation cohort comprised chest pain patients with negative cTn within 3 hours after symptom onset. This study has been approved by the ethics committee at these two hospitals. Written informed consent was obtained from all participants.

### 2.2 Patients enrollment

Any patient aged 18 or older, with acute nontraumatic chest pain occurring within the past 24 hours, and with negative initial cTn testing, was consecutively recruited.

Acute symptoms of myocardial ischemia, such as upper extremity, mandibular, or epigastric discomfort, or an ischemic equivalent such as dyspnea or fatigue, were also considered as chest pain according to the American Heart Association case definitions.^16^ Feelings of actual pain in anatomical chest area or any symptom that indicated myocardial ischemia were identified as generalized chest pain in this study.

Negative cTn indicated that the value of initial testing was under the 99th percentile upper reference limit (URL), specific to the assay used by each site. The contemporary cTnI assays arranged by emergency physicians in their daily work were used to determine the eligibility of enrollment in the derivation and validation cohorts.

Patients were excluded if they had ST‐elevation myocardial infarction (STEMI), or they were unable or unwilling to provide informed consent.

### 2.3 Data collection and measurements

Data collection was prospectively conducted on a standardized case report form by trained research assistants, according to the standard definitions for each candidate variable from a published data dictionary.^16^ Clinical symptoms were recorded as reported by the patient, and “no” was selected if certain situation was absent or unknown. Detailed information about symptom complex included eight sections, namely character, location, radiation, severity, time course, associated symptoms, precipitating factors, and relieving factors (Table 1). Pain severity was quantified by using the numeric rating scale (NRS), integer from 0 for no pain to 10 for worst pain.^17^ Heavy pain indicated the pain with NRS ≥ 5. Nocturnal onset indicated that the most significant symptoms occurred during the period from 8 pm to 8 am.

**Table 1:** Baseline characteristics of the derivation cohort (n = 1434)

		Univariable logistic regression analysis		
	n (%)	β coefficient	*P* value	OR (95%CI)
Age (y), mean (SD)	63.6 ± 13.7	0.028	0.001	1.029 (1.011, 1.046)
Male	684 (47.7)	0.306	0.155	1.358 (0.890, 2.072)
BMI (kg/m^2^), mean (SD)	25.1 ± 3.6	−0.069	0.025	0.933 (0.879, 0.991)
Risk factors				
Current smoker	217 (15.1)	0.849	<0.001	2.337 (1.449, 3.769)
Diabetes	320 (22.3)	0.546	0.018	1.726 (1.096, 2.717)
Hypertension	836 (58.3)	0.382	0.092	1.465 (0.939, 2.285)
Hyperlipidemia	126 (8.8)	−0.354	0.413	0.702 (0.300, 1.639)
Family history of premature CAD	233 (16.2)	−0.504	0.141	0.604 (0.309, 1.182)
Medical history				
Angina	645 (45)	−0.085	0.693	0.918 (0.601, 1.403)
MI	282 (19.7)	0.439	0.072	1.551 (0.961, 2.501)
Catheterization with stenosis ≥50%	358 (25)	−0.276	0.298	0.759 (0.452, 1.275)
PCI	282 (19.7)	−0.257	0.376	0.774 (0.438, 1.366)
CABG	25 (1.7)	0.693	0.268	1.999 (0.587, 6.804)
Heart failure	28 (2)	0.562	0.365	1.755 (0.520, 5.922)
CRF	12 (0.8)			
PAD	2 (0.1)			
Stroke	205 (14.3)	0.317	0.257	1.372 (0.794, 2.374)
Aspirin in recent 1 wk	715 (49.9)	−0.017	0.937	0.983 (0.646, 1.497)
Symptoms				
Characteristics				
Squeezing	112 (7.8)	0.712	0.025	2.039 (1.096, 3.793)
Crushing	44 (3.1)	0.055	0.928	1.057 (0.321, 3.480)
Heaviness	205 (14.3)	0.802	0.001	2.229 (1.366, 3.640)
Burning	56 (3.9)	0.363	0.451	1.437 (0.559, 3.692)
Distending	80 (5.6)	0.651	0.080	1.917 (0.926, 3.968)
Spasm	54 (3.8)	−0.607	0.405	0.545 (0.131, 2.273)
Stabbing	223 (15.6)	−0.707	0.061	0.493 (0.235, 1.032)
Sharp	20 (1.4)			
Tearing	8 (0.6)			
Aching	13 (0.9)			
Location				
Substernal	396 (27.6)	0.784	<0.001	2.190 (1.429, 3.356)
Left chest	507 (35.4)	−0.73	0.005	0.480 (0.289, 0.798)
Right chest	48 (3.3)	−1.206	0.235	0.299 (0.041, 2.194)
Across chest	68 (4.7)	0.535	0.196	1.708 (0.758, 3.847)
Epigastrium	49 (3.4)	−0.502	0.491	0.605 (0.145, 2.531)
Left arm	5 (0.3)	2.283	0.013	9.805 (1.618, 59.416)
Neck	17 (1.2)	0.664	0.382	1.943 (0.438, 8.626)
Jaw	5 (0.3)	1.290	0.251	3.633 (0.402, 32.837)
Both arms	4 (0.3)			
Shoulders	16 (1.1)			
Radiation				
Radiation	517 (36.1)	−0.180	0.431	0.835 (0.533, 1.308)
Severity				
Heavy pain (NRS 5~10)	570 (39.7)	0.890	<0.001	2.435 (1.585, 3.742)
Time course				
Nocturnal onset	644 (44.9)	−0.321	0.146	0.725 (0.470, 1.118)
Persistent or last ≥20 min	1009 (70.7)	0.232	0.351	1.261 (0.775, 2.050)
Associated symptoms				
Diaphoresis	350 (24.4)	0.824	<0.001	2.279 (1.479, 3.511)
Nausea	220 (15.3)	0.582	0.023	1.789 (1.083, 2.953)
Vomiting	69 (4.8)	0.962	0.008	2.618 (1.292, 5.304)
Dyspnea	113 (7.9)	0.799	0.010	2.224 (1.215, 4.068)
Chest distress	515 (35.9)	0.176	0.421	1.193 (0.776, 1.834)
Precipitating factors				
None	892 (62.2)	0.366	0.116	1.442 (0.914, 2.277)
Exertional	312 (21.8)	−0.085	0.748	0.918 (0.546, 1.545)
Breathing or cough	31 (2.2)			
Posture	15 (1)			
Eating	23 (1.6)	0.790	0.209	2.202 (0.642, 7.550)
Relieving factors				
None	714 (49.8)	0.263	0.223	1.301 (0.852, 1.986)
Nitrates	163 (11.4)	0.048	0.885	1.049 (0.547, 2.013)
Rest	206 (14.4)	0.230	0.420	1.259 (0.719, 2.204)
Posture	3 (0.2)			

Abbreviations: BMI, body mass index; CABG, coronary artery bypass grafting; CAD, coronary artery disease; CI, confidence interval; CRF, chronic renal failure; MI, myocardial infarction; NRS, numeric rating scale; OR, odds ratio; PAD, peripheral arterial disease; PCI, percutaneous coronary intervention.

Follow‐up was conducted by trained research assistants through telephone interviews at 30 days after enrolment, to acquire information about major adverse cardiac events (MACE) and hospital attendances.

Calculation methods for the history and other variables of HEART score have been described in previous articles.^7^, ^13^, ^14^ Four history classification methods are shown in Supporting Information Table S1. These four methods provide more detailed information than other papers about which combinations of what variables are high‐risk and which are low‐risk. So, we translated these calculation methods into computer programs using SAS. ECG interpretation was conducted by two independent cardiologists blinded to the symptoms, cTn levels and events. And discrepancies were evaluated by a third cardiologist. The HEART score was determined by the SAS programs to guarantee the consistency and comparability of scoring.

### 2.4 Outcome

The primary outcome was the composite end point of death from all causes and AMI, including the index AMI and subsequent AMI, during the 30 days after presentation to the ED. Each event in MACE was independently adjudicated by two senior cardiologists of clinical events committee using all available clinical records according to the international standardized definitions,^11^, ^16^, ^18^ and discrepancies were evaluated by a third senior cardiologist. All medical records were collected to help confirm these events and local death registry was used to supplement the survival status of patients lost to contact. MI was defined as detection of the rise and/or fall of cardiac biomarkers (cTn) with at least one value above the 99th percentile of the URL and with symptoms or ECG or imaging changes indicative of new ischemia.^11^

### 2.5 Statistical analyses

Univariable logistic regression was used to determine whether each individual symptom characteristic helped to predict the 30‐day MACE. Odds ratios with 95% confidence intervals were reported. Variables with *P* value ≤0.2 were eligible to enter the stepwise multiple logistic regression. Separate multivariable logistic regression models were developed for each section of symptom profile. The variables with *P* < 0.05 in each section entered the next multiple logistic analysis and those with *P* < 0.05 were retained in the final model (Figure S1). To test whether the CPSS is the independent predictor for 30‐day MACE, we developed a multivariable logistic regression model covering other known predictors, including sex, age, risk factor and ECG. Calculation methods for the age, risk factor and ECG were according to the HEART score. The Hosmer–Lemeshow goodness‐of‐fit test was used to evaluate the goodness‐of‐fit of the CPSS. To assess the discrimination of the CPSS for 30‐day MACE, the area under the receiver‐operating characteristic curve (AUC) was calculated and compared to the c‐statistics of different history component classification approaches of the HEART score reported in previous studies.^13^, ^14^ The discrimination of the HEART score with CPSS replacing the history variable was compared to the standard HEART scores with different history measurements. Reclassification, including category‐free net reclassification improvement (NRI) and integrated discrimination improvement (IDI), was used to assess how well the CPSS improving predictions compared with old HEART scores.^19^ Sensitivities, specificities, negative predictive values, positive predictive values and likelihood ratios of different cut‐offs of the CPSS were calculated for further clinic use. External validity of the CPSS was assessed in the validation cohort. All statistical analyses were performed using SAS V.9.4 (SAS Institute Inc., Cary, North Carolina, USA) or MedCalc V.11.4.2.0 (MedCalc Software, Ostend, Belgium).

## 3 RESULTS

**Figure 1:**
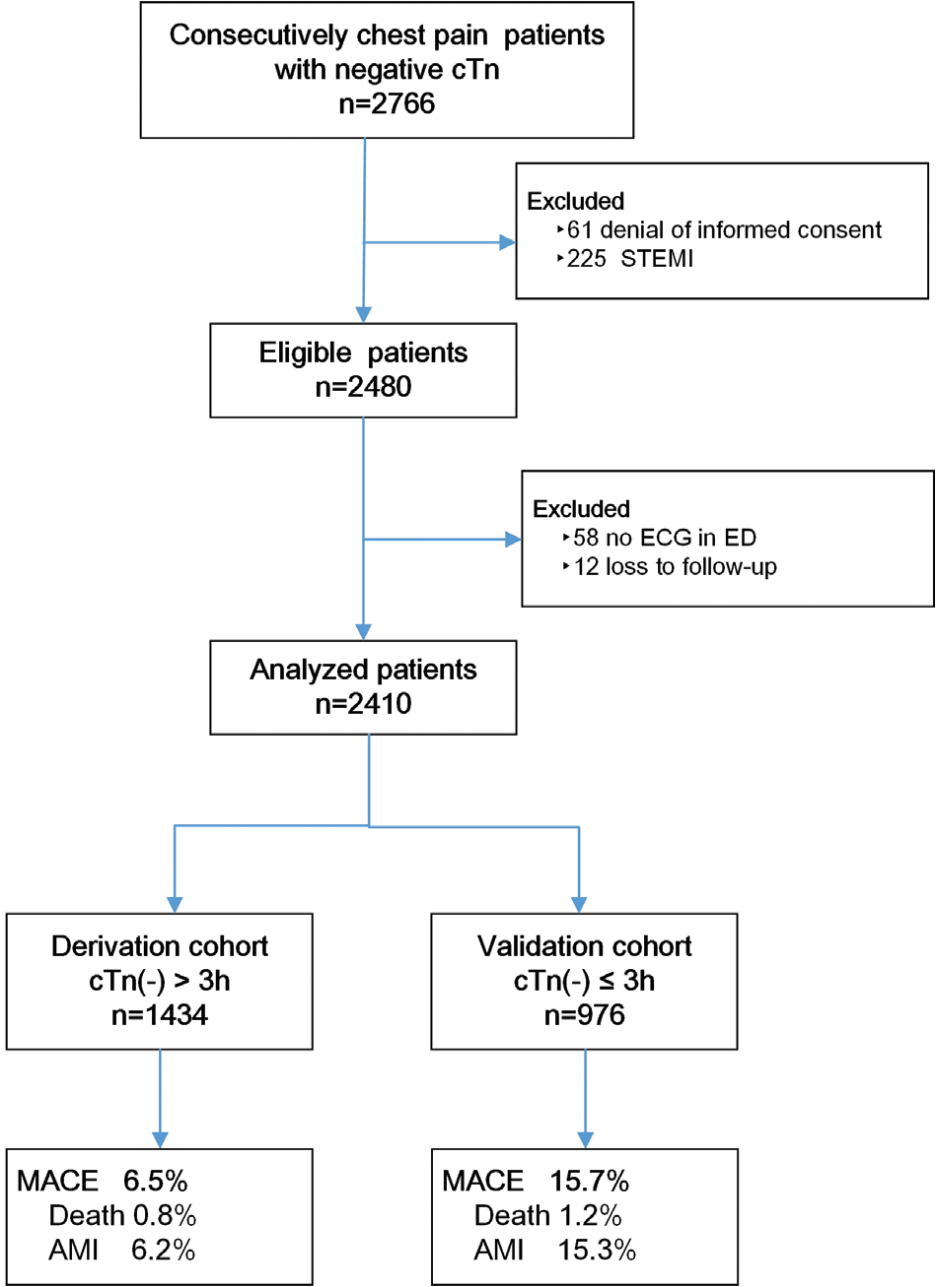
Study flowchart. AMI, acute myocardial infarction; cTn, cardiac troponin; ECG, electrocardiography; ED, emergency department; MACE, major adverse cardiovascular events; STEMI, ST‐segment elevation myocardial infarction

A total of 2766 patients with acute nontraumatic chest pain and negative initial cTn testing were consecutively recruited in participating hospitals from 24 August 2015 to 30 September 2017. Three hundred and fifty‐six patients were excluded for denial of informed consent (61), diagnosis of STEMI (225), deficiency of ECG (58), and loss to follow‐up (12). Finally, 2410 patients remained for derivation (1434) and validation (976) of the CPSS. There was no missing data regarding the presence or absence of symptom profiles in any patient. In derivation cohort, there were 93 (6.5%) patients with adjudicated MACE in 30 days after presentation. Twelve patients (0.8%) died from all causes and 89 (6.2%) were diagnosed with AMI (75 index AMI). And in validation cohort, MACE occurred in 153 (15.7%) patients, including 12 (1.2%) death and 149 (15.3%) AMI (142 index AMI) (Figure 1).

Demographic characteristics, risk factors, medical history, and detailed symptom profiles of patients in derivation cohort are shown in Table 1.

**Figure 2:**
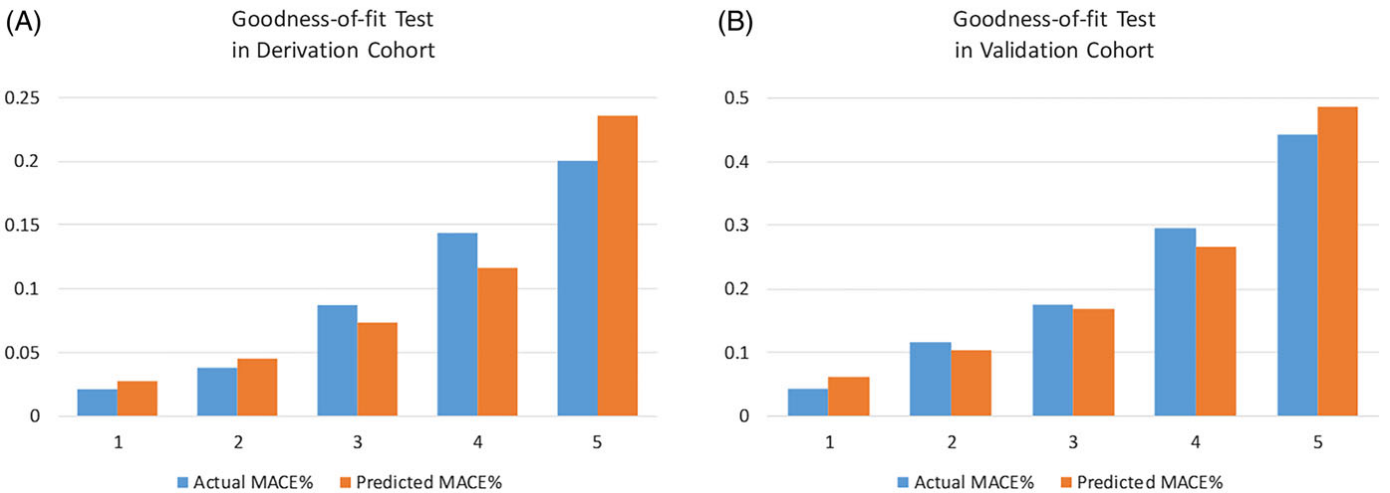
Hosmer–Lemeshow goodness‐of‐fit test for the CPSS model in the derivation (A, *P* = 0.30) and validation (B, *P* = 0.33) cohorts. CPSS, chest pain symptom score; MACE, major adverse cardiac event

Seven predictors in four aspects of chest pain symptom were identified after two stages of multivariable logistic regression with stepwise elimination (Table 2). For facilitated clinical use, integers were assigned for each of the seven predictors when they were present according to the relative numerical magnitudes of β coefficients. The CPSS was calculated by summing all the values of the seven predictors and identified as independent predictor for 30‐day AMI or death of chest pain patients in addition to the sex, age, and ECG using multivariable regression analysis (Table 2). The expected and observed event rates were proved to be well calibrated using the Hosmer–Lemeshow test (*P* = 0.30) (Figure 2A).

**Table 2:** Multivariable logistic regression analyses of predictors in the derivation cohort

	Multivariable logistic regression analyses			
	β Coefficient	*P* value	OR (95%CI)	CPSS
Character				
Heaviness	0.598	0.023	1.817 (1.086, 3.042)	1
Location				
Substernal	0.481	0.048	1.617 (1.004, 2.605)	1
Left arm	2.776	0.003	16.057 (2.517, 102.455)	5
Severity				
Heavy pain	0.594	0.017	1.811 (1.111, 2.954)	1
Associated symptoms				
Diaphoresis	0.641	0.006	1.898 (1.208, 2.982)	1
Vomiting	0.809	0.032	2.247 (1.072, 4.708)	2
Dyspnea	0.837	0.009	2.309 (1.233, 4.325)	2
CPSS (per 1 value)	0.509	<0.001	1.663 (1.433, 1.930)	
Male	0.475	0.039	1.607 (1.025, 2.520)	
Age	0.568	0.006	1.765 (1.181, 2.638)	
Risk factor	0.103	0.535	1.109 (0.800, 1.536)	
ECG	0.735	<0.001	2.085 (1.510, 2.879)	

Abbreviations: CI, confidence interval; CPSS, chest pain symptom score; ECG, electrocardiograph; OR, odds ratio.

**Figure 3:**
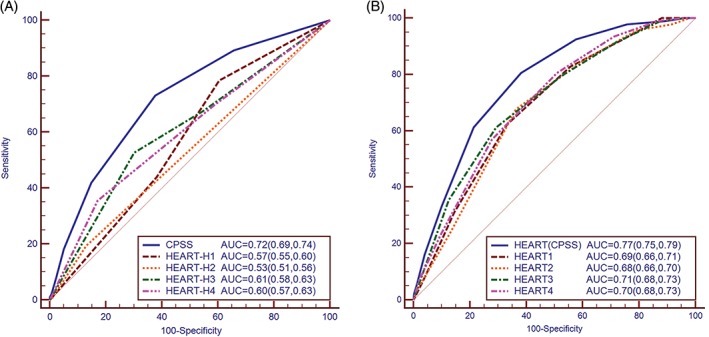
C‐statistics for the CPSS, history components (A) and HEART scores (B) in the derivation cohort. AUC, area under the receiver‐operating characteristic curve; CPSS, chest pain symptom score; HEART, history, ECG, age, risk factors, troponin; HEART‐H1~H4, different history component classifications of the HEART score; HEART1~4, HEART scores with different history component classifications; HEART(CPSS), the HEART score with the CPSS replacing history component

The CPSS performed well in the derivation cohort with the c‐statistics at 0.72 (0.69, 0.74), significantly better than the different history classifications (H1~H4) of the HEART score (*P* < 0.001) with values at 0.57, 0.53, 0.61, and 0.60 separately (Figure 3A).

The HEART score with CPSS replacing the history variable executed greater discrimination (AUC) than the standard HEART scores with different history measurements (*P* < 0.001) (Figure 3B). And both NRI and IDI showed that the new model with CPSS resulted in significant improvement in predicting performance (*P* < 0.001) (Table 3).

**Table 3:** The added predictive ability of the CPSS to the HEART scores in the derivation cohort

	NRI				IDI			
HEART(CPSS) vs Old model	NRI	SE	*z*‐score	*P* value	IDI	SE	*z*‐score	*P* value
HEART1	0.660	0.104	6.152	<0.001	0.039	0.009	4.342	<0.001
HEART2	0.669	0.100	6.243	<0.001	0.041	0.008	5.096	<0.001
HEART3	0.515	0.099	4.802	<0.001	0.030	0.007	3.973	<0.001
HEART4	0.619	0.095	5.771	<0.001	0.032	0.008	4.077	<0.001

Abbreviations: CPSS, chest pain symptom score; HEART, history, ECG, age, risk factors, troponin; HEART1~4, HEART scores with different history component classifications; HEART(CPSS), the HEART score with the CPSS replacing history component; IDI, integrated discrimination improvement; NRI, net reclassification improvement.

In the validation cohort, the CPSS performed well in calibration (*P* = 0.33) (Figure 2B), and the discrimination was also excellent with an AUC reaching 0.73 (0.70, 0.76). In addition, CPSS improved the HEART discriminatory capacity and risk classification with a significant increase in c‐statistic value, NRI, and IDI (Table 4).

**Table 4:** The added predictive ability of the CPSS to the HEART scores in the validation cohort

	AUC		NRI		IDI	
	AUC	*P* value	NRI	*P* value	IDI	*P* value
CPSS	0.73 (0.70, 0.76)					
HEART (CPSS)	0.79 (0.77, 0.82)					
HEART(CPSS) vs Old models						
HEART1	0.72 (0.69, 0.75)	<0.001	0.598	<0.001	0.078	<0.001
HEART2	0.72 (0.69, 0.74)	<0.001	0.642	<0.001	0.081	<0.001
HEART3	0.75 (0.72, 0.77)	<0.001	0.597	<0.001	0.051	<0.001
HEART4	0.76 (0.73, 0.79)	0.018	0.371	<0.001	0.044	<0.001

Abbreviations: AUC, area under the receiver‐operating characteristic curve; CPSS, chest pain symptom score; HEART, history, ECG, age, risk factors, troponin; HEART1~4, HEART scores with different history component classifications; HEART(CPSS), the HEART score with the CPSS replacing history component; IDI, integrated discrimination improvement; NRI, net reclassification improvement.

## 4 DISCUSSION

In this study, we developed a CPSS to assist in the prediction of 30‐day AMI or death in acute undifferentiated chest pain presenting to the ED with negative initial cTn over 3 hours after symptom onset. The CPSS served as an independent predictor and performed excellent in both the derivation and validation cohort. Moreover, the CPSS combined with the HEART risk score improved the discriminatory capacity of the old model and allowed the classification of risk levels significantly better. To our knowledge, this is the first attempt to derivate and validate the statistical combination of symptom features for diagnosis or prediction of AMI/death in chest pain patients.

This CPSS compromised seven predictors in four aspects of chest pain symptom. The substernal pain, left arm pain, diaphoresis, and vomiting were shown to be significant predictors of AMI in accordance with other studies.^20^, ^21^, ^22^ Some chest pain characteristics considered as “high‐risk” in the HEART score were found to have no significant diagnostic or prognostic value, like exertional pain and relief with nitroglycerin. Besides, the reported “low‐risk” variables^21^ (stabbing/sharp character, influenced by posture/breathing/cough, etc.) were also shown to be of no importance.

Using the same measurements with the history variable of HEART score, the CPSS improved the discrimination and risk classification significantly. In general, the symptom variables were integrated into a predictive score or decision rule for clinical use.^7^, ^9^, ^23^, ^24^, ^25^ Symptom features were usually analyzed as isolated variable with risk factors, medical history, ECG, and cTn in the multivariable logistic regression and the ones with significant importance were selected into the final model.^8^, ^9^, ^25^ However, there are some questions, whether single symptom variable was at the same level as ECG when using the multivariable regression analysis? Whether the organic integration of all aspects of symptom serve better than the isolated ones? Several studies have tried to establish a combinatorial chest pain score as one variable in the prediction model. However, the detailed development method and separate validation were not mentioned.^23^, ^26^, ^27^ Therefore, in order to portrait the symptom profile from all aspects (character, location, severity, radiation, time course, and so on), we develop the CPSS by using two‐step multivariable logistic regression. Interestingly, all of the variables except heaviness character in CPSS were identified as “typical” features in the HEART score,^13^, ^14^ and the new model overperformed the previous classification methods of history variable in the HEART score. This offered an opportunity to take a new look at the value of the combination of symptoms, but not single symptom in diagnose or prediction.

For risk stratification of chest pain in ED, the CPSS could add predictive information to the negative cTn as an independent predictor. Consistent with the kinetic profile of cTn releasing,^15^ it is too early to detect troponin rise within 3 hours, leading to misdiagnosis of AMI. The occurrence rate of AMI in patients with negative cTn within 3 hours after symptom onset was significantly higher than the rate in patients with negative cTn over 3 hours in this study. Undoubtedly, cTn should be retested later because of the relatively high misdiagnosis rate within 3 hours. But negative cTn over 3 hours after symptom was not safe enough, and there were still 6.5% major events missed, which was unacceptable for discharge. In the era of high sensitivity‐cTn (hs‐cTn), rule‐in and rule‐out of AMI in undifferentiated chest pain have been much more effective and safer than before.^4^ But there are still some actual reasons for considering the effective use of clinical information. Firstly, recent observations have questioned whether the European Society of Cardiology (ESC) 3‐hour pathway only using the hs‐cTn to rule in and rule out AMI provides adequate diagnostic performance.^28^ And data from one study has shown that the addition of clinical risk scores would improve the safety of pathways for early rule‐out AMI.^29^ Importantly, symptoms are the key components in these risk scores. Secondly, the accessibility of hs‐cTn is insufficient around the world currently, especially in the developing countries. Some in the Asia‐Pacific tertiary centers still use contemporary standard assays.^30^ As a result, since using hs‐cTn is still a development trend, simple and economic diagnostic tools are indeed needed now, such as the rational use of symptoms. In our study, the CPSS improved the discrimination and risk classification of risk model significantly, providing important pretest probability and reminding physicians to interpret results of laboratory testing more carefully. Furthermore, the performance of CPSS remained excellent in patients with negative cTn within 3 hours, demonstrating the external utilization potentiality in this population.

### 4.1 Limitations

This study also had several limitations. Firstly, the CPSS was developed from the ED chest pain patients in two hospitals of Shandong province in China. Although urban and rural hospitals were both covered and external validation was carried out in the same hospitals, the external generalization of the CPSS to wider patients should be determined by further studies in heterogeneous groups. Secondly, the HEART score was a very good reference substance here, but whether the CPSS‐HEART should be used in clinical practice needs more assessments. Since symptom merely plays a certain part of roles in the diagnosis/prognosis of chest pain, the integration with other significant predictors is prerequisite, such as demographic characteristics, medical history, risk factors, ECG, and cardiac markers. The combination of these components and the weights for selected variables require many explorations. Thirdly, considering that the sensitivity of the hs‐cTn is different from the contemporary cTn in patients assessed within 3 hours after onset of symptoms, we will take further evaluations based on the central testing of the hs‐cTn to assess the performance of the CPSS.

## 5 CONCLUSIONS

The CPSS performed well to assist in the prediction of 30‐day AMI or death in acute undifferentiated chest pain presenting to the ED with negative initial cTn, better than the different symptom classification methods of the HEART score. And the HEART score with CPSS replacing the history variable executed greater discrimination and reclassification than the standard HEART scores. This demonstrated that the effective combination of isolated variables was meaningful to make the most diagnostic or predictive value of symptoms and made preparations for the development of complete stratification models with medical history, risk factors, symptoms, ECG, and troponins for chest pain triage.

## CONFLICT OF INTERESTS

The authors declare no potential conflict of interests.

### Author contributions

Yuguo Chen, Jiali Wang, and Feng Xu contributed to the initiation, planning, and conduction of the study. Wen Zheng, Jingjing Ma, Guangmei Wang, He Zhang, and Jiaqi Zheng conducted the study supervision. Wen Zheng and Shuo Wu did the analysis and interpretation of data. Wen Zheng drafted the manuscript.

## Supporting information


**Figure S1.** Flowchart for developing the chest pain symptom score using logistic regressions.

**Table S1.** Four history classification methods of the HEART score.
