# Supplementary figures and images for: Effective combination of isolated symptom variables to help stratifying acute undifferentiated chest pain in the emergency department
Source: Clin Cardiol. 2019 Mar 19;42(4):467–75. doi: 10.1002/clc.23170 (PMC6712332; doi:10.1002/clc.23170)

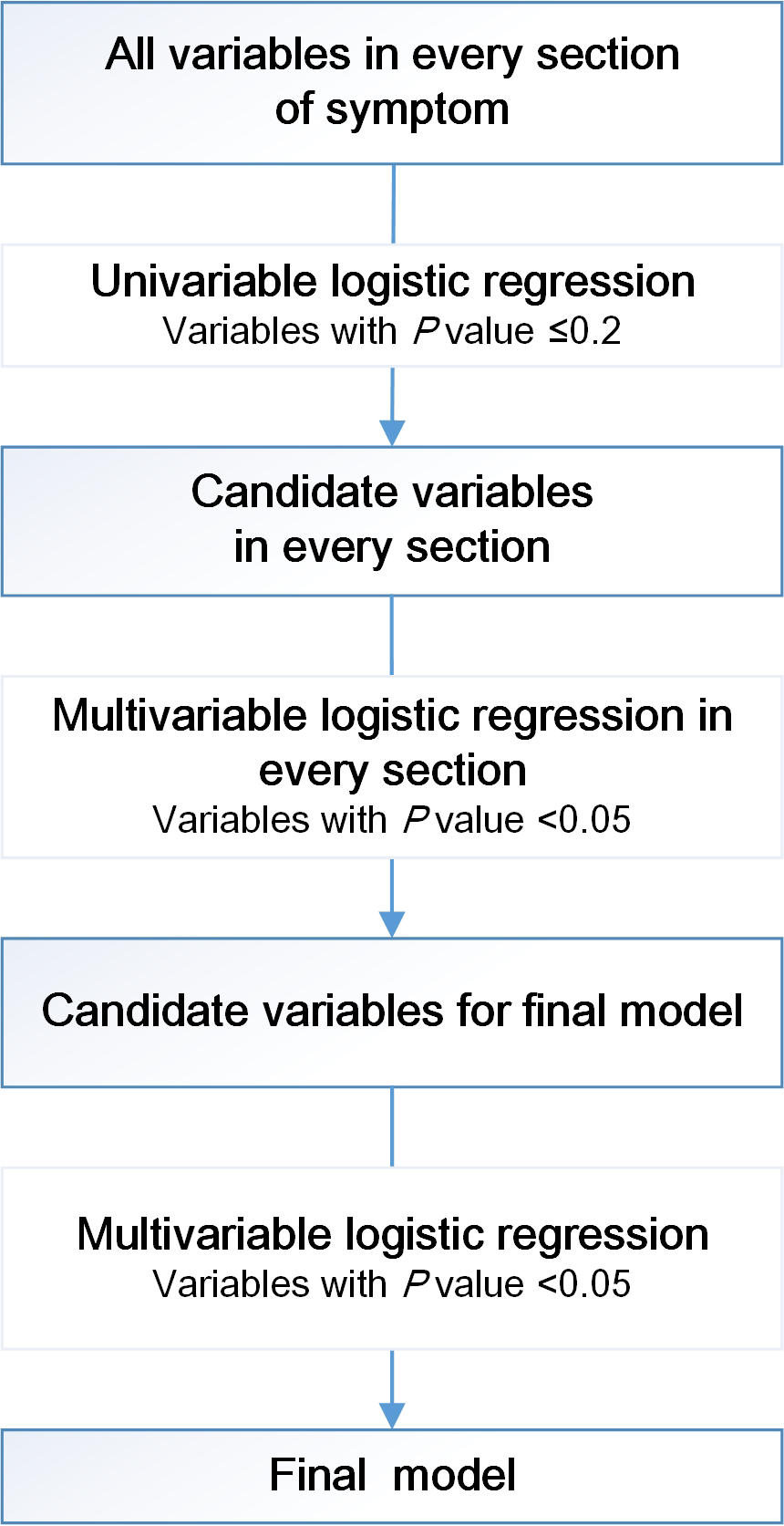

Supplement: Supplementary file 1 — Figure S1. Flowchart for developing the chest pain symptom score using logistic regressions. [file CLC-42-467-s001.tif]
