# Supplementary material for: Effective combination of isolated symptom variables to help stratifying acute undifferentiated chest pain in the emergency department
Source: Clin Cardiol. 2019 Mar 19;42(4):467–75. doi: 10.1002/clc.23170 (PMC6712332; doi:10.1002/clc.23170)
Supplement: Supplementary file 2 — Table S1. Four history classification methods of the HEART score. [file CLC-42-467-s002.docx]

**Supplementary materials**

**Table A.** Four history classification methods of the HEART score.

| **H1** | **H2** | **H3** | **H4** |
| --- | --- | --- | --- |
| 0 points: | 0 points: | 0 points: | 0 points: |
| Atypical only | 0–2 symptoms | 1–2 minor only | Mostly low-risk features |
| 1 point: | 1 point: | 1 point: | 1 point: |
| Typical and atypical | 3–4 symptoms | >1 major or >2 minor | Mixture of high-risk and low-risk features |
| 2 points: | 2 points: | 2 points: | 2 points: |
| Typical only | >4 symptoms | >1 major or major + minor | Mostly high-risk features |
| Typical: | Symptoms: | Major: | High-risk: |
| – Substernal chest pain | – Substernal chest pain | – Dyspnea | – Middle or left sided |
| – Exertional chest pain | – Exertional chest pain | – Substernal chest pain | – Heavy chest pain |
| – Relief with nitroglycerin | – Relief with nitroglycerin | – Exertional chest pain | – Diaphoresis |
| – Cold sweats/diaphoresis | – Weakness | – Relief with nitroglycerin | – Radiation |
| – Pain in L arm | – Nausea/vomiting | Minor: | – Nausea/vomiting |
| – Jaw pain | – Dyspnea | – Weakness | – Exertional |
| Atypical: | – Cold sweats/ diaphoresis | – Nausea/vomiting | – Relief of symptoms by sublingual nitrates |
| – Weakness | – Jaw/teeth pain | – Cold sweats/diaphoresis | Low-risk: |
| – Nausea | – Neck pain | – Jaw/teeth pain | – Well localized |
| – Dyspnea | – Pain in both arms | – Neck pain | – Sharp pain |
| – Dizziness | – Pain under shoulders | – Pain in both arms | – None-exertional |
| – Pain in both arms | – Pain in L arm | – Pain under shoulders | – No diaphoresis |
| – Pain under shoulders | – Dizziness | – Pain in L arm | – No nausea/vomiting |
| – Pain in R arm/shoulder | – Pain in R arm/shoulder | – Dizziness |  |
| – Neck pain |  | – Pain in R arm/shoulder |  |
